# The *PocketPerio* application significantly increases the accuracy of diagnosing periodontal conditions in didactic and chairside settings

**DOI:** 10.1038/s41598-024-59394-9

**Published:** 2024-05-03

**Authors:** Karo Parsegian, David K. Okano, Sangeetha Chandrasekaran, Yoolim Kim, Tonia C. Carter, Neel Shimpi, Sadaf Fadakar, Nikola Angelov

**Affiliations:** 1 Division of Periodontics, Department of Diagnostic Sciences and Surgical Dentistry, School of Dental Medicine, University of Colorado Anschutz Medical Campus, 13065 E 17Th Ave, Rm 130J, Aurora, CO 80045-2532 USA; 2 Department of Periodontics and Dental Hygiene, School of Dentistry, University of Texas Health Science Center at Houston, Houston, TX USA; 3 Section of Periodontics, School of Dentistry, University of Utah, Salt Lake City, UT USA; 4 Center for Precision Medicine Research, Marshfield Clinic Research Institute, Marshfield, WI USA; 5 Center for Dental Benefits, Coding and Quality, American Dental Association, Chicago, IL USA; 6 Predoctoral Dental Student, School of Dental Medicine, University of Colorado Anschutz Medical Campus, Aurora, CO USA

**Keywords:** Classification, Dental students, Diagnosis, Educational technology, Periodontics, Software, Periodontitis, Dental clinical teaching

## Abstract

The study aimed to determine the accuracy of diagnosing periodontal conditions using the developed web-based *PocketPerio* application and evaluate the user’s perspective on the use of *PocketPerio*. First, 22 third-year dental students (DS3) diagnosed ten cases without *PocketPerio* (control) and with *PocketPerio* (test) during a mock examination. Then, 105 DS3, 13 fourth-year dental students (DS4), and 32 senior second-year International Standing Program students (ISP2) used *PocketPerio* chairside. Statistical analysis was performed using a non-parametric paired two-tailed test of significance with the Wilcoxon matched-pairs signed rank test. The null hypothesis that *PocketPerio* did not increase the accuracy of periodontal diagnoses was rejected at α < 0.01. Periodontal diagnoses made using *PocketPerio* correlated with those made by periodontics faculty (“gold standard") in all cases. During the mock examination, *PocketPerio* significantly increased the accuracy of periodontal diagnoses compared to the control (52.73 vs. 13.18%, respectively). Chairside, *PocketPerio* significantly increased the accuracy of primary (100 vs. 40.0%) and secondary (100 vs. 14.25%) periodontal diagnoses compared to the respective controls. Students regardless of their training year felt more confident in diagnosing periodontal conditions using *PocketPerio* than their current tools, provided positive feedback on its features, and suggested avenues for its further development.

## Introduction

In 2017, the joint Workshop of the American Academy of Periodontology (AAP) and the European Federation of Periodontology (EFP) proposed the updated classification of periodontal and peri-implant diseases and conditions^1^. The classification introduced several key changes to definitions of periodontitis aimed to better reflect both biological and clinical disease dimensions with the intended use in clinical practice, clinical research, and epidemiologic studies^1–3^. These changes included but were not limited to (i) combining “chronic” and ”aggressive” phenotypes of periodontitis into a single “periodontitis” disease entity; (ii) defining periodontitis as the presence of interproximal clinical attachment loss (CAL) in ≥ 2 non-adjacent teeth or ≥ 3 mm buccal or lingual CAL in ≥ 2 teeth associated with inflammatory periodontal breakdown, and (iii) introducing the concepts of staging (reflecting the severity of periodontal breakdown) and grading (reflecting the rate of periodontal breakdown progression). Based on the severity, periodontitis was classified as stage I (initial periodontal breakdown—defined as a combination of 1-2 mm CAL and 4 mm pocket depth, PD), stage II (moderate periodontal breakdown – defined as a combination of 3-4 mm CAL and 5 mm PD), and stages III/IV (severe periodontal breakdown – defined as a combination of ≥ 5 mm CAL and ≥ 6 mm PD). Based on the progression rate, periodontitis was classified into grades A (the slow rate of progression), B (the moderate rate of progression), and C (the rapid rate of progression). The proposed updates were reflected in patient characteristics and the diagnostic accuracy of periodontal conditions^4^ and demonstrated a similar or greater diagnostic accuracy compared to diagnostic determinants proposed by the Community Periodontal Index and AAP together with the Centers for Disease Control and Prevention (CDC)^5–9^.

Accepting and using the updated classification in daily practice has been a continuous process, especially due to the presence of various “gray zone” clinical scenarios with overlapping clinical and radiographic findings^10–12^. A retrospective study using referral letters showed that although most patients (85%) were diagnosed using the updated disease definitions, the accuracy of periodontal diagnosis was relatively low (50.7 and 57.3% agreement for staging and grading, respectively)^13^. Other recent studies have shown that inter-participant agreement was 68.7–76.6, 75.5–82.0, and 82.4–84.8% for periodontitis staging, grading, and extent, respectively^14,15^. However, the agreement with all three diagnosis components (stage, grade, and extent) was low (47.2%)^16^. Furthermore, dental providers with limited exposure to periodontics found a differential diagnosis of periodontal conditions using the updated classification even more challenging compared to periodontists^17,18^. In addition, disparities in preexisting didactic and clinical knowledge can influence the performance of trainees diagnosing periodontal conditions chairside. Therefore, it is essential for dental educators to comprehensively understand the classification and effectively teach future dental providers to apply it chairside. This, in turn, requires the development of a straightforward approach to recognize and perform a differential diagnosis of periodontal conditions.

Several AAP/EFP Workshop-based diagrams and tables were developed to allow for a better understanding of clinical determinants of periodontal conditions and ensure their accurate diagnosis^1,2^. Several studies have described illustrative charts to improve the accuracy of periodontal diagnosis^19–21^. In our previous study, predoctoral dental students provided positive feedback on the use of the flowcharts and suggested developing a software application that would use a similar decision-tree approach to diagnose a wide variety of periodontal conditions^21^. Therefore, the goals of the present study were three-fold: (i) to address the needs of the educational community by developing a software application as a tool to assist in diagnosing a wide spectrum of periodontal conditions, (ii) to determine its accuracy in diagnosing periodontal conditions, and (iii) to evaluate the feedback of application users.

## Methods

### Ethics

All experimental protocols were approved by the Committee for the Protection of Human Subjects of the Institutional Review Board (IRB) of the University of Texas Health Science Center at Houston, School of Dentistry (UTSD, protocol #HSC-DB-18–0663 from October 28, 2020) and Colorado Multiple IRB of the University of Colorado Anschutz Medical Campus, School of Dental Medicine (CUSDM, protocol #22–2206 from January 6, 2023). For the mock examination held in March 2021 and to provide feedback, all students consented to participate in the respective activities by following respective invitation links. For the chairside use of *PocketPerio* in January–May 2023, an Information sheet/Postcard consent was used to obtain verbal consent since the IRB found that all criteria for waving the documentation of consent were met. The study was conducted in accordance with relevant IRB guidelines and regulations and principles of the 1975 Declaration of Helsinki, as revised in 2013^22^. The results were reported according to the 2016 Standards for Reporting of Diagnostic Accuracy Studies statement^23^.

### Technical specifications of *PocketPerio*

*PocketPerio* is a web-based application developed leveraging the Flutter software development kit and the Dart programming language. The implementation of both Flutter and Dart allows for *PocketPerio* portability across different platforms and web applications and the simplification of the application content and code updates. For future updates, *PocketPerio* is built to take advantage of update tools within the Flutter and Dart architecture, allowing for the classification changes to be implemented promptly. *PocketPerio* can be used on any mobile device running Android (version 4.1.x and above) or iOS (version 8 and above) and any device running Google Chrome, Microsoft Edge, Mozilla Firefox, or Safari web browsers. The decision-tree structure allows for a more expanded set of guidelines or amendments to the current guidelines to be inserted without incurring significant development time or overusing valuable development resources.

**Figure 1:**
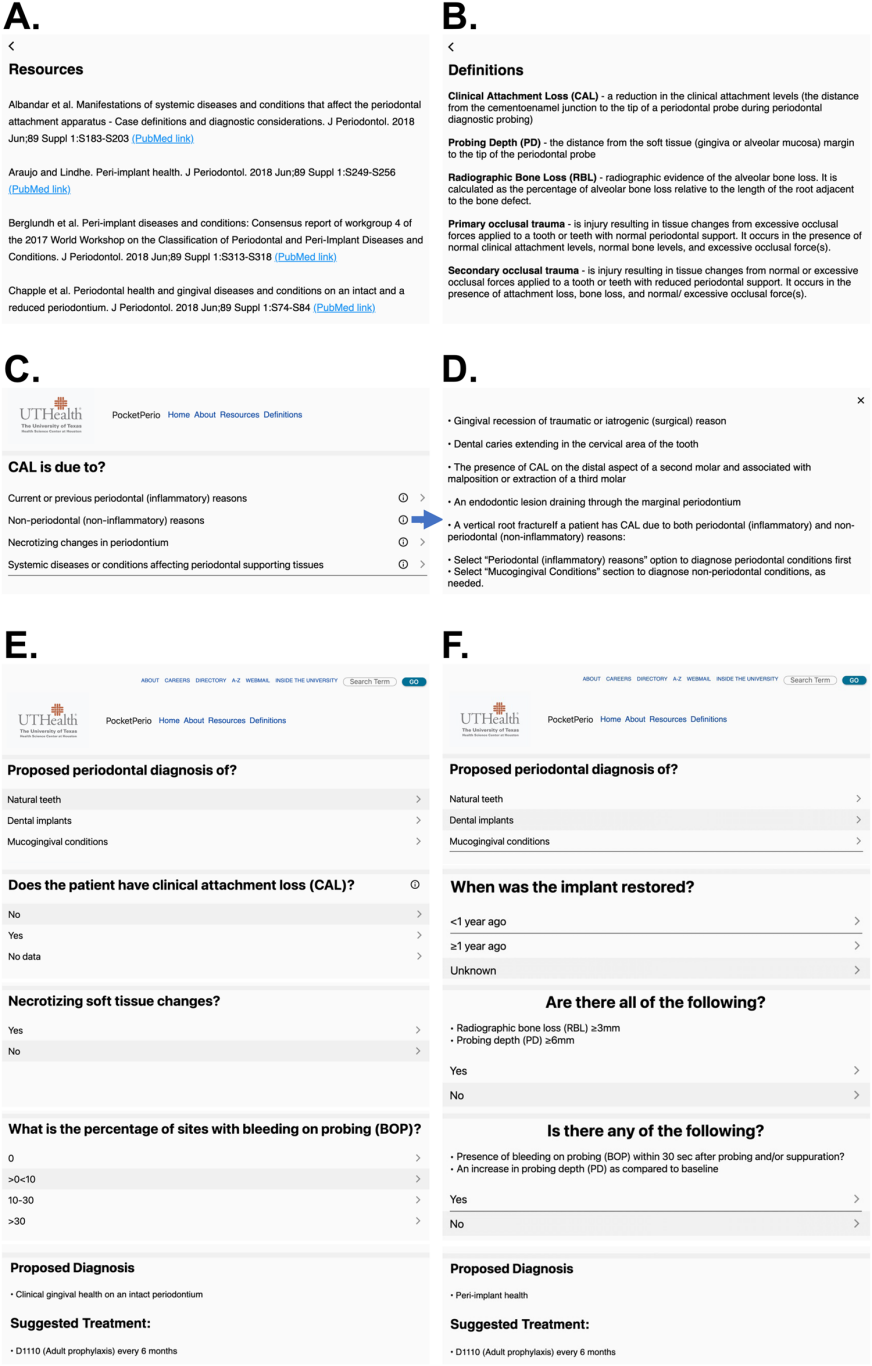
The interface and features of *PocketPerio*. **(A)** The “Resources” section of the application includes the list of peer-reviewed publications used to develop the decision-tree algorithm of questions and answers. **(B)** The “Definitions” section includes definitions of the most common conditions and abbreviations used throughout the application. **(C)** One of the main pages of the application that guides a user to answer questions. **(D)** The information icons contain a more detailed explanation and supportive material that helps users accurately understand the questions and choose the most appropriate answer. **(E)** and **(F)** Representative questions and answers are shown that lead to the diagnosis of clinical gingival health on an intact periodontium and peri-implant health, respectively. Note that each diagnosis follows by the suggested treatment options.

The questions and answer choices in the decision-tree algorithm of *PocketPerio* were based on the published Workshop guidelines in the “Resources” section of the application (Fig. 1A). In addition to a proposed periodontal diagnosis, *PocketPerio* includes definitions of periodontal terms (Fig. 1B), information icons with further explanations to clarify questions and answer choices (Fig. 1C and D), and suggested treatment options. Representative decision-tree approaches to diagnose clinical gingival health on intact periodontium and peri-implant health are shown in Fig. 1E and F, respectively. The process of arriving at the diagnosis of generalized periodontitis stage IV grade C and tooth- and prostheses-related factors is shown in Video 1.

### Eligibility criteria

The following participants met the inclusion criteria: (i) predoctoral dental students providing patient care at the UTSD and CUSDM and who (ii) completed didactic periodontics course(s) that taught the 2017 periodontal classification, and (iii) consented to participate in the study.

### Study design and methodology

The first part represented a crossover study conducted among UTSD third-year dental students (DS3) in the form of a mock examination/diagnosis. All participants received an online invitation to participate in the study and were informed that their decision to participate in the study was voluntary and would have no impact on their academic performance. The consented students (*n* = 22) took a classroom examination that consisted of ten clinical periodontal cases described previously^21^. The participants first diagnosed periodontal conditions using their curriculum-based knowledge and any available curriculum-based study materials including but not limited to lecture notes, decision-tree flowcharts^21^, and Workshop-based tables and diagrams^24^ that served as reference standards (*control*). Immediately after that, the participants diagnosed the same clinical cases using *PocketPerio* (*test*). Students recorded their answers, and the duration of their examination was recorded; however, no other identifiers were recorded. The elapsed time was not suspended once the participants started the examination. The participants were also asked to provide optional anonymous feedback on the use of *PocketPerio*.

The second part of the study represented a longitudinal study conducted among CUSDM DS3, fourth-year dental students (DS4), and 32 senior second-year International Standing Program students (ISP2) performing clinical chairside periodontal diagnosis of new patients who presented for the comprehensive periodontal examination. The patients had no history of periodontal treatment. The participants were asked to diagnose periodontal conditions using any available curriculum-based study materials that served as reference standards (*control*). Immediately afterwards, they were asked to diagnose periodontal conditions using *PocketPerio* (*test*). The same student could see more than one patient, but no patient was diagnosed more than once throughout the study. The accuracy of chairside diagnosis was calculated as the ratio between the diagnosis made without *PocketPerio* to that made using *PocketPerio*. Periodontics faculty supervising students' patient care also recorded their diagnosis(es) that served as reference “gold standards.” All faculty were calibrated through a series of departmental calibration sessions on the periodontal classification. A five-point Likert scale was used to evaluate the chairside participant’s responses to the following two questions: “*How difficult was it for you to diagnose periodontal conditions without PocketPerio?”* and “*How difficult was it for you to diagnose periodontal conditions with PocketPerio?”* Possible answers included “very easy/score 5”, “easy/score 4”, “moderate/score 3”, “difficult/score 2”, and “very difficult/score 1”). All responses were de-identified; however, the year of training was recorded for further stratification purposes. The survey was run using the Qualtrics software.

All students who used *PocketPerio* chairside were also asked to use Qualtrics software and provide additional anonymous feedback by answering six questions: “*How difficult was it for you to diagnose periodontal conditions without PocketPerio?*”, “*How difficult was it for you to diagnose periodontal conditions with PocketPerio?*”, “*What feature(s) of PocketPerio did you like the best?*”, “*What feature(s) of PocketPerio did you find not helpful?*”, “*If you were to make updates to PocketPerio, what would you change?*”, and “*Would you use PocketPerio in your practice?*” The participants were also allowed to add optional free-text comments. Although the responses were anonymous, the participants were asked to provide their years of training for further stratification purposes.

### Study outcomes

For the first part of the study (mock examination), the accuracy of periodontal diagnosis served as the study's primary outcome, and the duration of the examination was the secondary outcome. For the second (chairside) part of the study, the diagnostic accuracy of primary periodontal conditions served as the primary outcome. The diagnostic accuracy of secondary periodontal conditions and the duration of time needed to diagnose these conditions served as secondary outcomes. Periodontal conditions were stratified into primary and secondary diagnoses. Primary conditions included dental biofilm-induced periodontitis^2^. Secondary diagnoses included periodontitis as a manifestation of systemic diseases^24^, tooth- and prosthesis-related factors^25^, traumatic occlusal forces^26^, periodontal abscess^27^, and endodontic-periodontal lesions^27^.

### Null hypothesis

The use of *PocketPerio* did not increase the accuracy of diagnosing periodontal conditions.

### Power calculation

The determine the sample size, the MKPower program in R was used to simulate power for the Wilcoxon signed-rank test. First, a range of sample sizes from 10 to 100 was considered to estimate the range of sample sizes needed to achieve a power of at least 0.8. Second, power for the sample sizes within this range was calculated to identify the smallest sample size for obtaining a power of 0.8. The proportion of correct diagnoses was assumed to have a normal distribution with a mean of 20% and a standard deviation of 10%. In the first step, to consider a range of sample sizes, the sim.ssize.wilcox.test function in MKPower was used assuming that the true value of the difference in correct diagnoses between control and test conditions was 10%, the test was a two-sided paired test, sample sizes were between 10 and 100, and the significance level was 0.01. Simulations from 10,000 iterations resulted in a power of 0.47 for a sample size of 10 and a power of 0.92 for a sample size of 20. In the second step, to determine the minimum sample size needed for a power of 0.8, the sim.ssize.wilcox.test function in MKPower was used to calculate the power for sample sizes between 10 and 25, assuming the same parameters as in the first step. Simulations from 10,000 iterations showed that power ranged from 0.47 to 0.97 for these sample sizes. A power of 0.81 could be achieved with a sample size of 16, which was the minimum sample size considered for the study. In the simulations, a power of 0.95 could be obtained with a sample size of 22 participants.

### Statistical analysis

The comparison between control and test groups was performed using a non-parametric paired two-tailed test of significance with the Wilcoxon matched-pairs signed rank test. Statistical analysis was performed using GraphPad Prism 10 (GraphPad Software, San Diego, CA, USA), and the null hypothesis was rejected at α < 0.01. The word “significant” throughout the text refers to statistical significance.

## Results

### The use of *PocketPerio* during the mock examination

**Figure 2:**
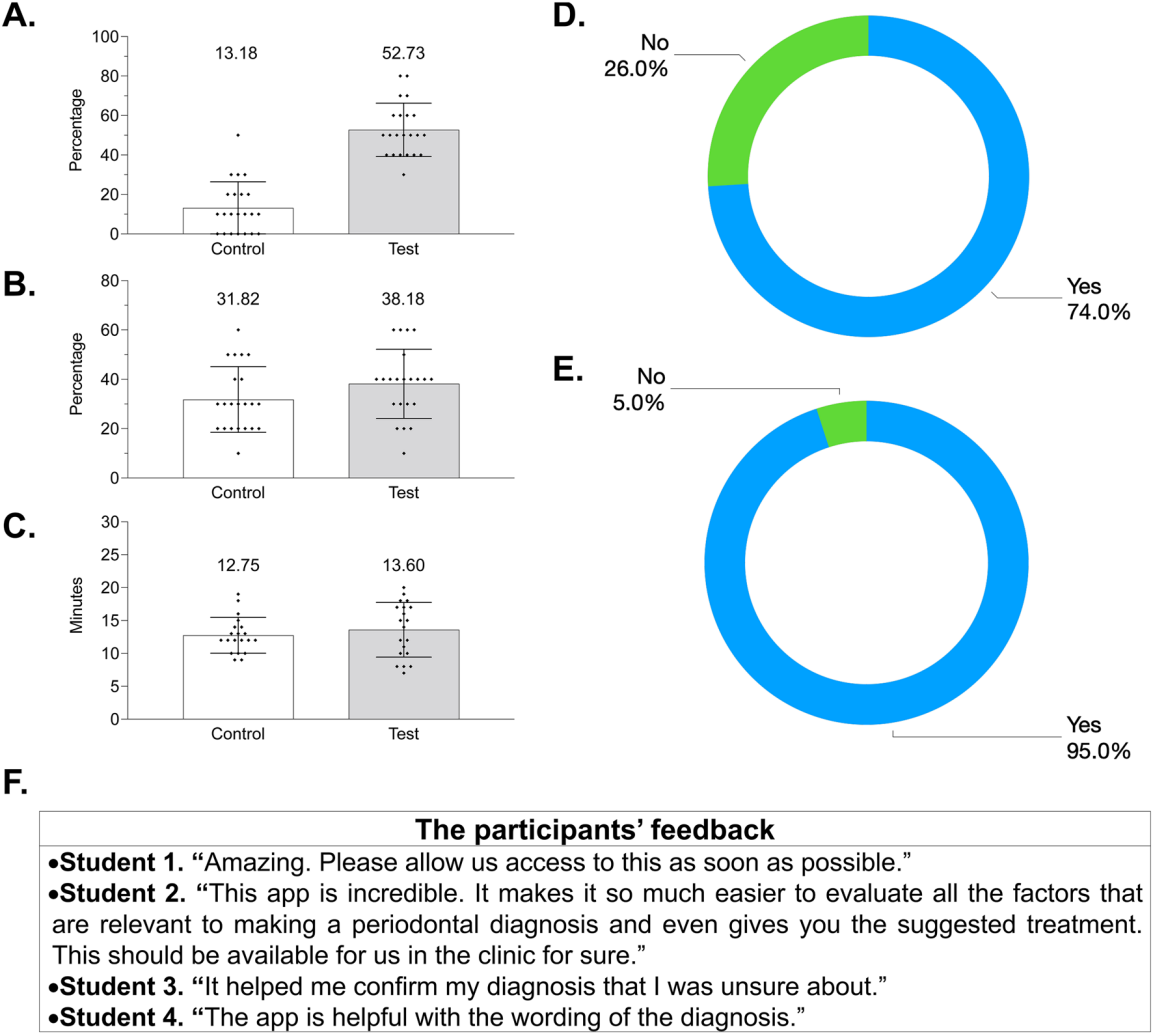
The accuracy and duration of the mock examination. DS3 who participated in the mock examination reviewed ten clinical cases first using their didactic knowledge and any additional tools (without *PocketPeri*o, *control*) and then using *PocketPerio* (*test*). The participants had a brief overview of *PocketPerio* before the examination. *PocketPerio* significantly improved the accuracy of diagnosing both primary and secondary diagnoses (52.73 ± 2.88 vs. 13.18 ± 2.82, respectively, *p* = 0.0000; Panel **A**); however, the accuracy of diagnosing secondary conditions (38.18 ± 2.99 vs. 31.82 ± 2.84, respectively, *p* = 0.103; Panel **B**) and the duration of the examination (13.60 ± 0.93 vs. 12.75 ± 0.61 min, respectively, *p* = 0.36; Panel **C**) were similar between the control and test groups. In Panels **A**-**C**, the numbers above each bar represent the respective percentages in each category. Most students (75%) were comfortable with diagnosing periodontal conditions using their didactic knowledge without *PocketPerio* (Panel **D**); however, almost all students (95%) were comfortable doing so using *PocketPerio* (Panel **E**). Several participants provided their feedback on the use of *PocketPerio* during the mock examination. Representative unedited comments are shown in Panel **F**. *DS3* Third-year dental students.

The mock examination was performed among UTSD DS3s in the middle of their academic year. The students were provided with instructions on how to use *PocketPerio* prior to the examination. Figure 2A shows that *PocketPerio* significantly increased the percentage of accurate diagnoses of both primary and secondary conditions (52.73 vs. 13.18%, respectively; *p* = 0.0000). No significant differences were observed between (i) test and control groups regarding the accuracy of diagnosing the primary condition when the diagnosis of the secondary condition was missing (38.18 vs. 31.82%, respectively; *p* = 0.10; Fig. 2B) and (ii) the duration of the examination (13.60 vs. 12.75 min, respectively; *p* = 0.36; Fig. 2C). Post-examination survey demonstrated that 75% of students were comfortable diagnosing periodontal conditions using their didactic knowledge and available supplemental resources (Fig. 2D). Almost all students (95%) were comfortable diagnosing periodontal diseases using *PocketPerio* (Fig. 2E). Several participants provided positive feedback on the use of *PocketPerio* (all responses are listed in Fig. 2F).

### The chairside use of *PocketPerio*

A total of CUSDM 150 students (105 DS3, 13 DS4, and 32 ISP2) participated in the chairside use of *PocketPerio*. Table 1 shows the frequencies of periodontitis diagnosed chairside. The most common diagnoses were generalized periodontitis stage IV grade C (43/150 cases, or 41.86%) followed by localized periodontitis stage III grades B and C (25/150 cases, or 16.67% for both diagnoses). No patients were diagnosed with generalized periodontitis stage II grade C, generalized periodontitis stage III grade A, generalized periodontitis stage IV grade A, and molar-incisor pattern periodontitis stage III grades A and B. When stratified by each diagnosis component separately, the most common disease extent was generalized followed by the localized and molar-incisor patterns (66.67, 32.67, and 0.67%, respectively). The most common stage was IV followed by III, and II (46.0, 42.0, and 12.0%, respectively). No patients were diagnosed with stage I (0%). Grade B was the most common one followed by grades C and A (52.67, 44.0, 3.33%, respectively).

**Table 1:** Primary periodontitis diagnoses made by predoctoral dental students chairside. DS3, DS4, and ISP2 who provided patient care during comprehensive periodontal evaluation were asked to diagnose periodontal conditions based on the full-mouth periodontal chart, recent (within the past 12 months) full-mouth radiographs, and both medical and dental history.

Primary periodontal diagnosis	Correct/total diagnosis *n*/*n* (%)			
	DS3	DS4	ISP2	Total
Localized periodontitis stage II grade A	0/1 (0)	0/0 (–)	0/0 (–)	0/1 (0)
Localized periodontitis stage II grade B	0/1 (0)	0/2 (0)	0/2 (0)	0/5 (0)
Localized periodontitis stage II grade C	0/1 (0)	0/0 (–)	0/0 (–)	0/1 (0)
Generalized periodontitis stage II grade A	0/1 (0)	0/0 (–)	0/0 (–)	0/1 (0)
Generalized periodontitis stage II grade B	1/6 (16.67)	0/0 (–)	3/4 (75.0)	4/10 (40.0)
Generalized periodontitis stage II grade C	0/0 (–)	0/0 (–)	0/0 (–)	0/0 (–)
Localized periodontitis stage III grade A	0/1 (0)	1/1 (100)	0/0 (–)	1/2 (50.0)
Localized periodontitis stage III grade B	10/15 (66.67)	2/2 (100)	5/8 (62.5)	17/25 (68.0)
Localized periodontitis stage III grade C	1/11 (9.09)	0/3 (0)	0/1 (0)	1/15 (6.67)
Generalized periodontitis stage III grade A	0/0 (–)	0/0 (–)	0/0 (–)	0/0 (–)
Generalized periodontitis stage III grade B	7/9 (87.5)	1/1 (100)	3/5 (60.0)	11/15 (73.33)
Generalized periodontitis stage III grade C	0/5 (0)	0/1 (0)	0/0 (–)	0/6 (0)
Generalized periodontitis stage IV grade A	0/1 (0)	0/0 (–)	0/0 (–)	0/1 (0)
Generalized periodontitis stage IV grade B	8/22 (36.36)	0/1 (0)	0/2 (0)	8/25 (32.0)
Generalized periodontitis stage IV grade C	13/30 (43.33)	1/2 (50)	4/11 (36.36)	18/43 (41.86)
Molar-incisor pattern periodontitis stage III grade A	0/0 (–)	0/0 (–)	0/0 (–)	0/0 (–)
Molar-incisor pattern periodontitis stage III grade B	0/0 (–)	0/0 (–)	0/0 (–)	0/0 (–)
Molar-incisor pattern periodontitis stage III grade C	0/1 (0)	0/0 (–)	0/0 (–)	0/1 (0)
Total	40/105 (38.1)	5/13 (38.46)	15/32 (46.88)	60/150 (40.00)

Students used their didactic knowledge and any available online and printed supplemental resources except *PocketPerio* (*control*). Immediately after that, the same students used *PocketPerio* (*test*) to diagnose the same patients. The accuracy of *PocketPerio* was calculated relative to covering faculty responses and correlated in all 150 cases (100%). The diagnosis made without *PocketPerio* was accurate in 60 cases (40.00%). When stratified based on the year of training (DS3, DS4, and ISP2), accurate periodontal diagnoses were made in 38.1, 38.46, and 46.88% of cases by DS3, DS4, and ISP2, respectively. *DS3* Third-year dental students, *DS4* Fourth-year dental students, *ISP2* Second-year International Standing Program students.

Table 1 also shows that the overall accuracy of diagnosis was 40.00 and 100% for the control and test groups. For the control group, the highest accuracy was demonstrated in diagnosing generalized periodontitis stage III grade B (73.33% of correct diagnoses) and localized periodontitis stage III grade B (68.0% of correct diagnoses). The lowest accuracy was observed while diagnosing localized periodontitis stage III grade C (6.67% of correct diagnoses). When stratified based on the year of training, ISP2 students had the highest diagnostic accuracy among control group students, followed by DS4 and DS3 (46.88, 38.46, and 38.10%, respectively). For DS3, the easiest diagnoses were generalized and localized periodontitis stage III grade B (87.5 and 66.67% of accurate diagnoses, respectively). For DS4, the easiest diagnoses were localized periodontitis stage III grades A and C and generalized periodontitis stage III grade B (100% accuracy for all diagnoses).

All 150 patients (100%) presented with at least one secondary periodontal condition identified while using *PocketPerio*. Table 2 shows that tooth- and prostheses-related factors were the most common condition followed closely by occlusal trauma, mucogingival deformities and conditions around teeth, periodontitis as a manifestation of systemic diseases, endodontic-periodontal lesions, and periodontal abscess (86.0, 84.67, 30.67, 29.33, 11.33, and 8.67%, respectively). Without using *PocketPerio*, secondary periodontal conditions were noted in 54 cases (14.25%), and only in two cases they were as complete as with *PocketPerio*. Among them, mucogingival deformities and conditions around teeth were the easiest condition to diagnose followed by endodontic-periodontal lesions, occlusal trauma, periodontitis as a manifestation of systemic diseases, and tooth- and prosthesis-related factors (57.7, 11.11, 11.0, 8.16, and 3.88%, respectively). No cases of periodontal abscess were diagnosed. When stratified based on the year of training, frequencies of diagnosing secondary conditions were the highest among DS4 followed by ISP2, and DS3 (25.89%, 17.72, and 12.03%, respectively). It is important to note that even if the secondary condition was identified by control students, no diagnosis was verbalized precisely according to the 2017 classification.

**Table 2:** Frequencies of diagnosing secondary periodontal conditions by control predoctoral dental students.

Secondary periodontal diagnosis	Correct/total diagnosis *n*/*n* (%)			
	DS3	DS4	ISP2	Total
Tooth- and prosthesis-related factors	2/89 (2.25)	2/10 (20)	1/30 (3.33)	5/129 (3.88)
Occlusal trauma	7/80 (8.75)	3/10 (30)	3/28 (10.71)	13/118 (11.0)
Mucogingival deformities and conditions around teeth	18/36 (50)	3/4 (75)	9/12 (75.0)	30/52 (57.7)
Periodontitis as a manifestation of systemic diseases	4/34 (11.76)	0/5 (0)	0/5 (0)	4/49 (8.16)
Endodontic-periodontal lesions	1/16 (6.25)	0/0 (–)	1/2 (50.0)	2/18 (11.11)
Periodontal abscess	0/11 (0)	0/0 (–)	0/2 (0)	0/13 (0)
Total	32/266 (12.03)	8/29 (25.89)	14/79 (17.72)	54/379 (14.25)

All 150 patients had secondary periodontal conditions (a total of 379 diagnosis incidents), with tooth- and prosthesis-related factors and occlusal trauma being the most common condition (129 and 118 cases, respectively). Students diagnosed 54 out of 379 diagnosis incidents (14.25%). Among the conditions, mucogingival deformities and conditions around teeth were the most common ones to diagnose (57.7% of all diagnoses of these conditions). Among classes, DS4 had the highest proportion of diagnosing secondary conditions (25.89%), whereas DS3 had the lowest frequencies (12.03%). Secondary diagnoses correlated between *PocketPerio* and periodontics faculty responses in all cases. *DS3* Third-year dental students, *DS4* Fourth-year dental students, *ISP2* Second-year International Standing Program students.

### The participant’s feedback on the use of *PocketPerio*

The participants who used *PocketPerio* chairside were also asked to provide optional anonymous feedback on their comfort level of diagnosing periodontal conditions without and with *PocketPerio* (Supplementary Table 1). A total of 18 students (7, 6, and 5 DS3, DS4, and ISP2, respectively) completed the survey. The 5-point Likert scale was used to evaluate students’ comfort level while diagnosing periodontitis chairside without and with *PocketPerio*. Without *PocketPerio*, average scores were 3.86 (range 3–5), 4.2 (range 4–5), and 3.4 (range 2–5) for DS3, DS4, and ISP2, respectively (the average score for all classes was ~ 3.81). With *PocketPerio*, average scores were 4.29 (range 4–5), 4.5 (range 4–5), and 4.4 (range 3–5) for DS3, DS4, and ISP2, respectively (the average score for all classes was ~ 4.38).

The same students were also asked to comment on *PocketPerio* features (Supplementary Table 2). When asked, “*What feature(s) of PocketPerio did you like the best?*” fourteen students chose “The ability to accurately diagnose periodontal conditions,” nine students chose “The interface”, eleven students chose “The included proposed treatment options,” and eight students chose “The included information icons.” When asked, "*What feature(s) of PocketPerio did you find not helpful?*”, three students said that they found terminology and verbalization of questions and answers to be not as straightforward, one student found the interface to be not too easy to navigate, and another student felt that some features of *PocketPerio* were redundant (but did not provide additional detail). When asked, “*If you were to make updates to PocketPerio, what would you change?*”, most students asked to make a more advanced, machine-learning version of *PocketPerio.* Some students asked for updated terminology and verbalization of questions and answers, a simplified version of *PocketPerio* with fewer diagnostic choices, and an updated interface. When asked, “*Would you use PocketPerio in your practice?*”, fourteen students responded “Yes” and three students responded, “Maybe, if there is an updated version.” No student chose “No” as an answer. Some students also provided written comments outside the proposed answer options.

## Discussion

In the present study, we reported on the accuracy of the *PocketPerio* application in diagnosing various periodontal conditions in both classroom and clinical settings. Mock examination results showed that *PocketPerio* did not decrease the duration of time required for students to arrive at the diagnosis compared to the control. The participants were given only brief instructions on using *PocketPerio* before the mock examination, and it is expected that users using *PocketPerio* regularly will find it intuitive and spend less time arriving at the diagnosis. It is important to note that the application requires a manual selection of answers, the process of arriving at the diagnosis is not automatic and requires time to read questions, answer options, and make an accurate selection. At least to some extent, this lack of user familiarity using *PocketPerio* could explain why test students did not accurately diagnose all cases (the accuracy was 52.73%).

It is also important to highlight differences in the prevalence of severe periodontitis in patients attending academic dental clinics reported in the present study compared to the general U.S. population. In the present study, severe periodontitis corresponding to stages III and IV (defined as CAL ≥ 5 mm and PD ≥ 6 mm on ≥ 2 non-adjacent teeth based on the 2017 periodontal classification^1^) was 88.67% (42.67 and 46.0% for stages III and IV, respectively). A large-scale, 2009–2014 National Health and Nutrition Examination Survey-based study that included 10,683 adult patients (aged ≥ 30 years) showed that the prevalence of severe periodontitis (defined as ≥ 2 interproximal sites with CAL ≥ 6 mm on ≥ 2 non-adjacent teeth and ≥ 1 interproximal site with PD ≥ 5 mm, based on the 2012 AAP/CDC periodontitis case definition^28^) was 7.8% of all participants that corresponded to ~ 18.4% of all periodontitis cases^5^. Using the 2012 AAP/CDC periodontitis case definition^28^, the retrospective analysis of 10,544 electronic health records (EHRs) at East Carolina rural practice-based clinics demonstrated that the prevalence of severe periodontitis was ~ 35.0%^29^. Several other studies reported on the prevalence of severe periodontitis in an academic setting. The retrospective analysis of 2,137 EHRs at Western University (Pomona, CA, USA) showed that the prevalence of severe periodontitis (defined as CAL ≥ 5 mm based on the 1999 periodontal classification^30^) was 24.5%^31^. The retrospective analysis of 1,131 EHRs at Harvard University showed that the prevalence of severe periodontitis (defined as > 30% of root length or > 5 mm of alveolar bone loss based on the 2015 Task Force Report on the Update to the 1999 Classification^32^) was 2.8%^33^. Overall, differences in the prevalence of severe periodontitis among these studies (including the present study) could be due to different definitions and cut-offs of the disease and different study populations. The geospatial distribution of severe periodontitis throughout the United States was reported previously^34,35^ and should be considered. Although Colorado state was not among the states with the highest prevalence of severe periodontitis, some state counties had a high prevalence of the disease. To note, our unpublished data showed that the prevalence of stages III and IV periodontitis at the UTSD ranged from 50 to 84.6%.

Although the participants in the present study were able to accurately diagnose ~ 43% of periodontitis cases, some clinical cases posed significant challenges, resulting in decreasing the accuracy of periodontal diagnosis to as low as 0%. These data demonstrate a high variability in the diagnosis accuracy and highlight the need for tools like *PocketPerio* to assist and complement students’ knowledge. Another important finding of the study was that an overwhelming majority of secondary periodontal diagnoses (~ 85%) remained undiagnosed. Since all patients had at least one secondary diagnosis, it is essential to emphasize the importance of these conditions, especially while developing a comprehensive treatment plan. For example, studies demonstrated the added benefit of occlusal therapy in slowing the progression of periodontitis and improving therapeutic tooth prognosis. The presence of tooth- and prostheses-related factors (such as defective restorations) can be associated with the retention of dental biofilm and negatively impact periodontal conditions and tooth prognosis^36^. Although it is possible that students noted these and other secondary conditions during their periodontal evaluation, it is important to educate students to document them properly to teach them to be competent independent providers.

*PocketPerio* also increased the students’ confidence in accurately diagnosing periodontal conditions regardless of the end-user’s level of knowledge. For example, ISP2 were much less comfortable diagnosing periodontal conditions without *PocketPerio* than DS3 and DS4, whereas their confidence level became comparable while using *PocketPerio*. Importantly, during both mock examination and chairside, the participants were able to navigate through the application and use it even without previous knowledge of its interface and features. Overall, the students provided positive and encouraging feedback on the interface and features of *PocketPerio*; however, some changes were also suggested (updated terminology, improved verbalization of some questions and answers, and the development of a simplified application version).

Interestingly, most suggestions for future changes to *PocketPerio* were related to the development of its more advanced, machine-learning version. This suggestion reflects an emerging use of machine-learning-assisted approaches in the daily teaching process. Artificial intelligence (AI) and machine-learning-assisted approaches have been continuously implicated in dentistry to detect a wide variety of radiographic findings^37,38^, perform and analyze immune profiling to assist with the diagnosis of peri-implantitis^39^, enhance the interdisciplinary interaction between medical and dental professionals^40^, and assist with diagnosis of periodontitis^41^. Nevertheless, limitations in study design and the associated high risk of bias make the use of these applications in dental practice challenging^42,43^. The development of an AI version of *PocketPerio* will be considered in the future. It is encouraging that almost all responders to the anonymous survey said they would use *PocketPerio* in their future clinical practice.

*PocketPerio* is not the first published application aimed at diagnosing periodontal conditions. A recent study reported that the Android-based PerioSmart application improved the accuracy of diagnosing periodontitis in a time-effective manner^44^. Although both PerioSmart and *PocketPerio* are aimed to improve the accuracy of periodontal diagnosis, there are a few differences between these applications. First, *PocketPerio* assists with the diagnosis of a wide scope of periodontal conditions and not only periodontitis. Additionally, *PocketPerio* provides suggested treatment options for each diagnosis. Finally, *PocketPerio* is a web-based application, which is available on any device connected to the Internet and is not limited to a specific operating system. The free access to *PocketPerio* can be requested by contacting the corresponding author.

There are several strengths of the study. First, it is the development of a novel educational application that we expect to meet several essential educational goals and has the potential to be converted into educational enduring material. This accomplishment would help maintain educational standards and patient care in both academic and private clinical settings. Second, the study was performed in two dental schools and included researchers from several other research institutions, which allows for more intense inter-institutional collaboration and, to some extent, generalization of the results. In addition, the participants of the study represent dental students of various years of training, including those trained internationally. Finally, the study provides a foundation for further updates of *PocketPerio* and possibly creating an AI version of this tool.

There are several limitations of the present study. *PocketPerio* was used only by dental students, and therefore its effectiveness among other dental providers (such as general dentists and other dental specialists) remains to be explored. The scope of periodontal conditions encountered in predoctoral clinics was quite limited (for example, there were no cases of generalized periodontitis stage II grade C, generalized periodontitis stage III grade A, and molar-incisor pattern periodontitis except stage III grade C); therefore, the effectiveness of *PocketPerio* to diagnose conditions rarely encountered in clinical practice remains to be explored. The participants’ feedback on *PocketPerio* and their perception of its interface could, at least to some extent, be influenced by their year of training (and, consequently, by the level of knowledge of the periodontal classification) and the frequency of using the application. Since the mock examination and chairside use of *PocketPerio* were performed in only a single school (UTSD and CUSDM, respectively) and sample sizes were modest (*n* = 22 and 150, respectively), our results may not allow for their generalization. Finally, sample sizes varied in different experimental approaches. For example, the number of participating DS4 was ~ 10 times lower than DS3, which could influence study outcomes and interpretation of the data.

## Conclusions

*PocketPerio* offers a straightforward approach to improving the accuracy of diagnosing a wide spectrum of periodontal conditions, especially among users with limited exposure to periodontics.

### Supplementary Information

Supplementary Information 1.

Supplementary Video 1.

## Data Availability

The raw datasets used and/or analyzed during the current study are available from the corresponding author upon reasonable request and subject to clearance by the respective IRB of the UTSD (Houston, TX, USA) and the CUSDM (Aurora, CO, USA).
