# Supplementary material for: The PocketPerio application significantly increases the accuracy of diagnosing periodontal conditions in didactic and chairside settings
Source: Sci Rep. 2024 May 3;14:10189. doi: 10.1038/s41598-024-59394-9 (PMC11068793; doi:10.1038/s41598-024-59394-9)
Supplement: Supplementary file 1 — Supplementary Information 1. [file 41598_2024_59394_MOESM1_ESM.docx]

**Supplementary information**

| **Survey question** | **DS3**  **(*n* = 7)** | **DS4**  **(*n* = 4)** | **ISP2**  **(*n* = 5)** | **Total**  **(*n* = 16)** |
| --- | --- | --- | --- | --- |
|  | **Control (no *PocketPerio*)** | | | |
| Extremely difficult (score 1) | 0 | 0 | 0 | 0 |
| Somewhat difficult (score 2) | 0 | 0 | 1 | 1 |
| Neither difficult nor easy (score 3) | 3 | 0 | 2 | 5 |
| Somewhat easy (score 4) | 2 | 3 | 1 | 6 |
| Extremely easy (score 5) | 2 | 1 | 1 | 4 |
| Mean ± STDEV | 3.86 ± 0.9 | 4.2 ± 0.45 | 3.4 ± 1.14 | 3.81 ± 0.91 |
|  | **Test (*PocketPerio*)** | | | |
| Extremely difficult (score 1) | 0 | 0 | 0 | 0 |
| Somewhat difficult (score 2) | 0 | 0 | 0 | 0 |
| Neither difficult nor easy (score 3) | 0 | 0 | 1 | 1 |
| Somewhat easy (score 4) | 5 | 2 | 1 | 8 |
| Extremely easy (score 5) | 2 | 2 | 3 | 7 |
| Mean ± STDEV | 4.29 ± 0.44 | 4.50 ± 0.58 | 4.40 ± 0.89 | 4.38 ± 0.62 |

**Supplementary Table 1.** The self-evaluation of participant’s knowledge to diagnose periodontal conditions chairside. Students were asked to provide optional feedback on their skills to diagnose periodontal conditions without *PocketPerio* (*control*) and with it (*test*). Seven DS3, four DS4, and five ISP2 (a total of 16 students) completed the questionnaire. *PocketPerio* increased the confidence of students to diagnose periodontal conditions, especially among ISP2 students, compared to the control (4.4 vs. 3.4, respectively). The evaluation was performed using the five-point Likert scale. Results represent the mean ± standard deviation (STDEV).

| **Survey questions** | **Answer options** | **DS3**  **(*n* = 7)** | **DS4**  **(*n* = 6)** | **ISP2**  **(*n* = 5)** | **Total**  **(*n* = 18)** |
| --- | --- | --- | --- | --- | --- |
| What features of *PocketPerio* did you like the best? | - The ability to assist with a diagnosis. - Interface. - Suggested treatment options. - The included information icons. | 7  4  5  2 | 4  2  3  2 | 3  3  3  4 | 14  9  11  8 |
| What feature(s) of *PocketPerio* did you find not helpful? | - Interface. - Terminology and verbalization of questions and answers. - Redundant features. | 0  2  0 | 0  0  0 | 1  1  1 | 1  3  1 |
| If you were to make updates to *PocketPerio*, what would you change? | - Change the interface. - Make a simplified version with fewer diagnostic choices. - Update the terminology and verbalization. - Make a more advanced, machine-learning version. | 0  1  1  5 | 1  1  0  1 | 1  1  2  2 | 2  3  3  8 |
| If you had a chance to use *PocketPerio* in your practice, would you do so? | - No. - Maybe, if there is an updated version. - Yes. | 0  1  7 | 0  1  3 | 0  1  4 | 0  3  14 |
| Optional anonymous comments | - **Student 1.** “Selection process to navigate accurately was difficult.” - **Student 2.** “The times I have used *PocketPerio*, it was very simple and straightforward, and I was not confused at all. It is very informative, and it would be extremely helpful to have in the clinic.” - **Student 3.** “When we make a diagnosis, sometimes we are between 2 choices, so *PocketPerio* helps you confirm or guide you in another direction. It's a great addition to diagnosing our patients.” - **Student 4.** “I would surely use *PocketPerio* in my future practice, as the new classification is still confusing to a lot of us. I think it would help a lot with CONFIRMING that our diagnosis is correct.” - **Student 5.** “I think it is a great app!! Super easy to use!” - **Student 6.** “I like how it walks you through the diagnosis so no etiologies factors are missed.” - **Student 7.** “It’s very effective at diagnosis.” - **Student 8.** “It was helpful to confirm but I always got the same diagnose.” | | | | |

**Supplementary Table 2.** The participants’ feedback on the use of *PocketPerio* chairside. Test DS3, DS4, and ISP2 students who used *PocketPerio* were also asked to provide optional anonymous feedback on the most and least useful features of *PocketPerio* as well as on changes that they would like to see. A total of 18 students (7 DS3, 6 DS4, and 5 ISP2) completed the survey. Overall, the participants provided positive feedback on their experience using *PocketPerio* and expressed an interest in using it in their future clinical practice. Some students provided constructive criticism on the *PocketPerio* interface and the way some decision-tree questions were verbalized.
